# Treatment outcomes of 130 patients underwent an endoscopic discectomy

**Published:** 2012-11

**Authors:** Mehdi Abili, Behzad Naruie, Reza Akhavan Sigari

**Affiliations:** ^*a*^Department of Neurosurgery, Taleghani Hospital, Medical University of Mashhad, Mashhad, Iran.; ^*b*^Ali Ebne Abitaleb Hospital ,Medical University of Zahedan, Zahedan, Iran.; ^*c*^University Hospital of Gottingen Germany, Gottingen, Germany.

## Abstract

**Background:**

Endoscopic discectomy method is a novel technique that is increasingly used in spine surgery. Previous studies have reported that some common complications like dural adhesions are lower in this technique compared with the other techniques. Furthermore, treatment outcomes are reportedly higher because of minimal invasion. Thepresent study aims to determine the outcome of percutaneous endoscopic discectomy.

**Methods:**

A total of 130 patients underwent the lumbar disc prolapse operations during 2008 to 2012 in all of them the entire procedure was performed endoscopically. All procedures were carried out from a posterior approach using a 4-mm Hopkins 0 degrees-telescope placed in the working insert equipped with channels for suction tube, operative instruments and nerve root retractor (ENDOSPINE instrumentation (Karl STORZ GmbH and Co. KG). The pre- and post-operation pain was assessed using a Visual Analog Scale (VAS). Furthermore, the treatment outcome was assessed using modified MacNab criteria before operation, 24 hours, one month, 2 months, 6 months, one year, and two years after operation .

**Results:**

Good to excellent outcome was achieved in 89% of patients, which is comparable with the results of classic microdiscectomy. The mean age of patients was 35.6 years old and the mean length of follow-up was 3.4 years. There was significant reduction in the severity of back pain and lower limb symptoms at 6 months and 2 years post-operation. There was significant improvement in all aspects of the Quality of Life scores at 6 months and 2 years post-operation. In 3 patients the dural sac was lacerated but none of the tears was exceed a few mm in length with not association with neural injury.

**Conclusions:**

Findings of this study showed that percutaneous endoscopic lumbar discectomy is associated with improvement in back pain and lower limb symptoms. It has the advantage that it can be performed on a day case basis with short length of hospitalization and early return to work thus improving quality of life earlier.

**Keywords:**

Endoscopic discectomy, Spine surgery, Lumbar disc prolapse operation, Outcome


					Volume 4, Suppl. 1 Nov 2012
Publisher: Kermanshah University of Medical Sciences
URL: http://www.jivresearch.org
Copyright:
Congress of Iranian Neurosurgeons, 14-16 November 2012, Kermanshah, Iran
Paper No. 34
Treatment outcomes of 130 patients underwent an endoscopic
discectomy
Mehdi Abili a,*
, Behzad Naruie b
, Reza Akhavan Sigari c
a
Department of Neurosurgery, Taleghani Hospital, Medical University of Mashhad, Mashhad, Iran.
b
Ali Ebne Abitaleb Hospital ,Medical University of Zahedan, Zahedan, Iran.
c
University Hospital of Gottingen Germany, Gottingen, Germany.
Abstract:
Background: Endoscopic discectomy method is a novel technique that is increasingly used in spine surgery. Previous
studies have reported that some common complications like dural adhesions are lower in this technique compared
with the other techniques. Furthermore, treatment outcomes are reportedly higher because of minimal invasion.
Thepresent study aims to determine the outcome of percutaneous endoscopic discectomy.
Methods: A total of 130 patients underwent the lumbar disc prolapse operations during 2008 to 2012 in all of them
the entire procedure was performed endoscopically. All procedures were carried out from a posterior approach
using a 4-mm Hopkins 0 degrees-telescope placed in the working insert equipped with channels for suction tube,
operative instruments and nerve root retractor (ENDOSPINE instrumentation (Karl STORZ GmbH and Co. KG). The
pre- and post-operation pain was assessed using a Visual Analog Scale (VAS). Furthermore, the treatment outcome
was assessed using modified MacNab criteria before operation, 24 hours, one month, 2 months, 6 months, one year,
and two years after operation .
Results: Good to excellent outcome was achieved in 89% of patients, which is comparable with the results of classic
microdiscectomy. The mean age of patients was 35.6 years old and the mean length of follow-up was 3.4 years.
There was significant reduction in the severity of back pain and lower limb symptoms at 6 months and 2 years post-
operation. There was significant improvement in all aspects of the Quality of Life scores at 6 months and 2 years
post-operation. In 3 patients the dural sac was lacerated but none of the tears was exceed a few mm in length with
not association with neural injury.
Conclusions: Findings of this study showed that percutaneous endoscopic lumbar discectomy is associated with
improvement in back pain and lower limb symptoms. It has the advantage that it can be performed on a day case
basis with short length of hospitalization and early return to work thus improving quality of life earlier.
Keywords:
Endoscopic discectomy, Spine surgery, Lumbar disc prolapse operation, Outcome
* Corresponding Author at:
Mehdi Abili: Department of Neurosurgery ,Taleghani Hospital, Medical University of Mashhad, Mashhad, Iran. Tel: 09155126242,
Email: Dr_abili@yahoo.com, (Abili M.).

				

